# Identifying priorities for the collection and use of data related to community first response and out-of-hospital cardiac arrest: protocol for a nominal group technique study

**DOI:** 10.12688/hrbopenres.13347.1

**Published:** 2021-07-26

**Authors:** Dylan Keegan, Eithne Heffernan, Jenny McSharry, Tomás Barry, Siobhán Masterson

**Affiliations:** 1 Discipline of General Practice, Clinical Science Institute, School of Medicine, National University of Ireland, Galway, Galway, H91 TK33, Ireland; 2 Health Behaviour Change Research Group, School of Psychology, National University of Ireland, Galway, Galway, H91 TK33, Ireland; 3 School of Medicine, University College Dublin, Dublin 4, D04 V1W8, Ireland; 4 National Ambulance Service, Health Service Executive, St. Eunan's Hall, St Conal's Hospital, Letterkenny, Donegal, F92 XK84, Ireland

**Keywords:** first response, community first responders, out-of-hospital cardiac arrest, nominal group technique, prehospital emergency care, consensus meeting, priority list, outcome measurement

## Abstract

**Introduction:** Out-of-hospital cardiac arrest (OHCA) is a devastating health event that affects over 2000 people each year in Ireland. Survival rate is low, but immediate intervention and initiation of cardiopulmonary resuscitation (CPR) and administration of an automated external defibrillator (AED) can increase chances of survival. It is not always possible for the emergency medical services (EMS) to reach OHCA cases quickly. As such, volunteers, including lay and professional responders (e.g. off-duty paramedics and fire-fighters), trained in CPR and AED use, are mobilised by the EMS to respond locally to prehospital medical emergencies (e.g. OHCA and stroke). This is known as community first response (CFR).

Data on the impact of CFR interventions are limited. This research aims to identify the most important CFR data to collect and analyse, the most important uses of CFR data, as well as barriers and facilitators to data collection and use. This can inform policies to optimise the practice of CFR in Ireland.

**Methods:** The nominal group technique (NGT) is a structured consensus process where key stakeholders (e.g. CFR volunteers, clinicians, EMS personnel, and patients/relatives) develop a set of prioritised recommendations. This study will employ the NGT, incorporating an online survey and online consensus meeting, to develop a priority list for the collection and use of CFR data in Ireland. Stakeholder responses will also identify barriers and facilitators to data collection and use, as well as indicators that improvements to these processes have been achieved. The maximum sample size for the NGT will be 20 participants to ensure sufficient representation from stakeholder groups.

**Discussion:** This study, employing the NGT, will consult key stakeholders to establish CFR data collection, analysis, and use priorities. Results from this study will inform CFR research, practice, and policy, to improve the national CFR service model and inform international response programs.

## Introduction

Out-of-hospital cardiac arrest (OHCA) is a leading cause of mortality around the world ^1^. In Ireland, each year there are over 2000 OHCA where resuscitation is attempted ^2^. The survival rate from OHCA in Ireland is low (~ 7%) ^3^, but timely responses can increase the chance of survival. There is much geographic variation in Ireland, with people living in a range of geographical settings ^4^. This variation in accessibility can impact the response times of the emergency medical services (EMS) to OHCA calls. The prospect of survival from OHCA reduces by approximately 10% with every minute before resuscitation is started. Therefore, each minute is literally vital in the event of OHCA ^5^. Consequently, many countries, including Ireland, have implemented first response programmes. These are dispatch systems for the deployment of a rapid response team to cardiac arrests or other medical emergencies in the prehospital setting ^6, 7^. A range of responder groups (e.g. firefighters, police officers, citizen-responders, off-duty EMS personnel) can be dispatched. These first responders can work alongside the EMS, as part of the EMS, or instead of the EMS. They can be classified as volunteers or non-volunteers, with variations in training, equipment, and dispatch methods ^7^. There are wide variations in first response systems within and between countries, with some having multiple systems ^6^.

In Ireland, individuals and groups have provided a volunteer community response to OHCA for many years. These individuals, known as community first responders, are volunteers mobilised by the EMS to respond to prehospital medical emergencies (e.g. OHCA, stroke, and choking) in their locality. They can include lay responders and professional responders, such as off-duty paramedics, general practitioners, nurses, police officers, and fire-fighters ^6, 7^. They are often organised in teams within their community ^7^. They are typically trained in cardiopulmonary resuscitation (CPR) and automated external defibrillator (AED) use and can provide support to the EMS, patients and patients’ families when an OHCA call is made. They are sometimes the first to arrive at the scene, performing CPR and administering an AED. In addition, they can collect patient data, including current patient condition and past medical history, to provide to the EMS ^3^.

Data on community first response (CFR) in Ireland are currently limited. Therefore, the true impact of this intervention is difficult to estimate. As such, it is necessary to establish data collection and use priorities for CFR, including identifying key data that should be measured, collected, and reported as standard during emergency calls involving CFR. These data could be utilised to evaluate and provide evidence for CFR, and could inform policies that will optimise the practice of community response in Ireland. Traditionally, data collection for OHCA interventions has focused on the outcomes of return of spontaneous circulation (ROSC) during resuscitation and short-term patient survival (including at the scene, on arrival to hospital, and hospital discharge) ^8^. However, there are many other considerations during community response to cardiac arrest that lie outside of the binary outcome of patient survival/death. For instance, research suggests that CFR volunteers provide important support to patients’ families, as well as valuable training and resources to their community ^7^.

Currently, there are two core outcome sets (COSs) ^9^ specifying the outcomes that should be measured as a minimum during clinical trials of interventions for cardiac arrest survivors; Core Outcome Set for Cardiac Arrest (COSCA) ^10^ and Paediatric Core Outcome Set for Cardiac Arrest (P-COSCA) ^11^. Both COSs broaden the range of cardiac arrest outcomes to include not only survival but also neurological function, physical function, and health-related quality of life at various time points post-discharge. These outcomes provide a more balanced measurement of short- and long-term patient condition ^10, 11^. Research into COS development purports that a standardised approach to outcome measurement ^12^ can improve the value of research, consistency of measurements, ensure outcomes are relevant, and influence future research and practice, while reducing reporting bias, particularly in randomised trials ^13^. COSs clearly define a minimum set of data for measurements that are important to stakeholders, while highlighting that additional relevant outcomes can also be reported as required ^12^.

This study will adopt a similar approach to develop priorities for data collection and use, specifically focusing on CFR in Ireland. These priorities will be developed primarily for CFR practice, though they could also be used in CFR research. The priorities will not focus solely on OHCA management because, while this is an important component of CFR, volunteers in Ireland attend a wider range of emergencies, including stroke, heart attack, and choking. It is important to capture the rounded experience and outcomes of CFR, to understand and measure its impact, and improve the CFR service as a whole. This research will ensure that CFR data are collected in a standardised, consistent manner, which will help build an evidence base for CFR and inform improvements to policy and practice for this intervention. This research will also ensure that the data collected and the uses to which those data are put are valued by key stakeholders, such as clinicians, patients, and CFR volunteers. In addition to developing data collection and use priorities, this study will also identify barriers and facilitators to data collection and use during CFR.

The aim of this project is to consult a range of stakeholders, including CFR volunteers, EMS professionals, patients, clinicians, and researchers, on which CFR data are most important to collect and how information can be utilised to improve the community response service. Their responses will inform the development of a priority list for the collection and use of CFR data in Ireland. The specific objectives of this research are to identify:

(1) The most important data to record and analyse related to CFR.
(2) The most important uses of data related to CFR.
(3) Facilitators and barriers to the collection and use of data related to CFR.
(4) Indicators that improvements to the collection and use of data related to CFR have been achieved.

## Methods

### Design

The nominal group technique (NGT) is a process in which consensus is used to generate and rank ideas and prioritise topics. There are typically four stages involved in a NGT process: (1) silent generation, (2) round robin, (3) clarification, and (4) voting ^14^. Stage one involves the private generation of responses by participants, based on questions provided by the research team. Stage two involves participants sharing their responses with the wider group and the responses being recorded. Stage three involves small group discussions of the list of responses provided by the participants. Stage four involves private voting to prioritise and rank responses according to which responses the participants think are most important. The NGT is frequently used in health service research to identify and agree on priorities, for example identifying quality markers in general practice ^15^, evaluating exercise adherence measures in patients with musculoskeletal disorders ^16^, and determining important treatment outcomes for patients with aphasia ^17^. The process facilitates a balanced selection of ideas/topics that are important to the participant group as a whole ^18^.

In this study, the NGT will be conducted virtually, as current public health guidelines in Ireland do not allow large indoor face-to-face gatherings due to the COVID-19 pandemic. The NGT will facilitate idea generation and consensus development with a range of stakeholder groups, identifying core CFR data for collection and analysis, as well as the barriers and facilitators to this process. This method was chosen because it is an established technique for generating ideas and prioritising topics ^18^.

The specific NGT procedure used in this study was based on the procedure in a study that aimed to prioritise target behaviours for research on diabetes ^19^. It will entail two key components: (1) an online survey and (2) an online consensus meeting. The online survey will be used to gather information on the four study objectives. Specifically, it will be used to generate a comprehensive list of CFR data that should be recorded and analysed (Topic 1), as well as a comprehensive list of the uses to which these data should be put (Topic 2). In addition, the survey will be used to identify facilitators and barriers to the collection and use of CFR data (Topic 3), as well as indicators that improvements to these processes have been achieved (Topic 4). The consensus meeting will be used to identify priorities for the collection, analysis, and use of CFR data. In particular, the meeting will be used to develop a shortlist of the most important CFR data to record and analyse (Topic 1), as well as a shortlist of the most important uses of these data (Topic 2).

### Ethical considerations

Ethics approval has been granted by the Research Ethics Committee of the National University of Ireland (NUI) Galway (reference no. 18-Sept-13). This research will be conducted in accordance with NUI Galway’s University Data Protection Policy and Ireland’s General Data Protection Regulation (GDPR). All participants will provide written informed consent prior to the NGT process. The NGT employed here is a component of the third work package of a larger programme of research. Work package 1 examined the international context of community response to OHCA, work package 2 examined sources of community response data in Ireland, and work package 3 will examine data collection, analysis, and integration of community response data working with stakeholders to inform policy and practice ^7^.

### Project registration

This project involves the generation of a list of core data to be collected for the CFR intervention and has been registered on the Core Outcome Measure in Effectiveness Trials (COMET) website.

### Participants and recruitment

Participants will be recruited to represent various stakeholder groups, including CFRs, patients, patients’ families, clinicians, researchers, and policy-makers. This study involves multiple stakeholder groups and as such, to facilitate adequate representation from each cohort while maintaining a manageable size for the conduct of group discussions during the NGT process, the maximum sample size will be 20 participants ^19^. They will be invited to take part in both the survey and the meeting. Peer consultation amongst the research team and project collaborators will be employed to identify and recruit participants from stakeholder groups.

### Procedure

Each participant will be emailed a study invitation and a study information sheet. They will be given the opportunity to contact the research team with any questions. They will then provide written informed consent before completing the online survey, which will be generated through Microsoft Forms. In advance of the online consensus meeting, the participants will be sent copies of the comprehensive lists for Topic 1 and Topic 2, which will be generated based on the survey responses. The meeting will be held approximately one month after the survey is launched. The meeting will last approximately three hours and be facilitated by members of the research team using the video platform Zoom. The research team will first outline the format and aims of the meeting. Participants will then be divided into small groups to discuss the lists for Topic 1 and Topic 2, and to suggest any additional items that should be included in the lists, if needed. Subsequently, the participants will carry out a ranking exercise for Topic 1. Slido, an online polling tool, will be used to facilitate this exercise. Each participant will privately rank the items listed for Topic 1 to prioritise what they consider to be the most important data to collect and analyse. Slido will calculate the results of this ranking exercise for the group as a whole. All participants will then take part in a group discussion about the ranking results. Following this discussion, a second round of ranking will take place via Slido to finalise the priorities for Topic 1. This process will be repeated for the Topic 2 list, which is a comprehensive list of the uses to which data relating to CFR should be put. Finally, the research team will gather the participants’ views on the collection and use of data related to community response and OHCA.

### Analysis

Three stages of analysis will be utilised throughout the NGT process. Firstly, the responses from the pre-meeting online survey will be analysed by the research team in order to generate a comprehensive list of suggestions for each of the four topics included in the survey. The lists for Topic 1 and Topic 2 will be shared with the participants before and during the consensus meeting to facilitate discussion and ranking. Secondly, during the consensus meeting, participants will rank the suggestions from the Topic 1 and Topic 2 lists using the Slido tool. The tool will also calculate the overall ranking results for the group as a whole. The suggestions ranked highest will receive a score of 10, those ranked second will receive a score of 9, and so on. The sum of the points for each suggestion will be calculated and divided by the number of participants who completed the poll to generate an averaged, ranked score. The top scoring suggestions will be presented back to the participants for discussion.

### Public and patient involvement

A panel of three CFR volunteers, including lay and professional volunteers, have assisted with the development of study materials, including pilot testing the online participant survey. The panel also advised the research team on the NGT procedure, including providing feedback and guidance on the length of the meeting, adequate break times for participants, and ensuring balanced representation from each participant group.

### Plans for dissemination

The results of this project will be used to provide recommendations to the National Ambulance Service (NAS) and Prehospital Emergency Care Council (PHECC) of Ireland on CFR data collection, utilisation, and analysis, as well as identify the barriers and facilitators to this process. The findings will also be submitted for publication in peer-reviewed journals, presented at academic conferences, and shared with project collaborators and research participants.

### Study status

The study is ongoing and participant enrolment has permanently closed.

## Discussion

This project will provide an agreed upon set of priorities from a range of stakeholder groups regarding the most important CFR data to collect and their most important applications. The NGT will facilitate a virtual discussion with key stakeholders and allow for this priority list of key data to be chosen. The outcomes of this process will help provide evidence for the development of policy and practice to generate a sustainable, improved community response model in Ireland. This research will inform the national community response programme about which data are most important to collect and use during community response to OHCA, as well as barriers and facilitators to data collection and use. It will also inform international community response programmes of important outcomes and data to collect and analyse during community response to OHCA. There is currently no minimum set of data to measure during community response to OHCA, and this study will address this need.

## Data availability

### Underlying data

No underlying data are associated with this article.

### Extended data

Open Science Framework: Identifying priorities for the collection and use of data related to community first response and out-of-hospital cardiac arrest: protocol for a nominal group technique study, https://doi.org/10.17605/OSF.IO/VT5M7 ^20^

This project contains the following extended data:

- Study invitation and information sheet
- Consent form and research survey

Data are available under the terms of the Creative Commons Zero "No rights reserved" data waiver (CC0 1.0 Public domain dedication).

## References

[1] Myat A Song KJ Rea T : Out-of-hospital cardiac arrest: current concepts. *Lancet.* 2018;391(10124):970–979. 10.1016/S0140-6736(18)30472-0 29536861

[2] OHCAR: Out of Hospital Cardiac Arrest Register, Annual Report 2019. 2020. Reference Source

[3] Barry T Guerin S Bury G : Motivation, challenges and realities of volunteer community cardiac arrest response: a qualitative study of 'lay' community first responders. *BMJ Open.* 2019;9(8):e029015. 10.1136/bmjopen-2019-029015 31399458 PMC6701604

[4] Masterson S Cullinan J Teljeur C : The Spatial Distribution of Out-of-Hospital Cardiac Arrest and the Chain of Survival in Ireland: A Multi-Class Urban-Rural Analysis. *Irish Geography.* 2016;49(2):1–27. Reference Source

[5] Larsen MP Eisenberg MS Cummins RO : Predicting survival from out-of-hospital cardiac arrest: a graphic model. *Ann Emerg Med.* 1993;22(11):1652–8. 10.1016/s0196-0644(05)81302-2 8214853

[6] Oving I Masterson S Tjelmeland IBM : First-response treatment after out-of-hospital cardiac arrest: a survey of current practices across 29 countries in Europe. *Scand J Trauma Resusc Emerg Med.* 2019;27(1):112. 10.1186/s13049-019-0689-0 31842928 PMC6916130

[7] Heffernan E Mc Sharry J Murphy A : Community first response and out-of-hospital cardiac arrest: a qualitative study of the views and experiences of international experts. *BMJ Open.* 2021;11(3):e042307. 10.1136/bmjopen-2020-042307 33757945 PMC7993284

[8] Gräsner JT Wnent J Herlitz J : Survival after out-of-hospital cardiac arrest in Europe - Results of the EuReCa TWO study. *Resuscitation.* 2020;148:218–226. 10.1016/j.resuscitation.2019.12.042 32027980

[9] Webbe J Sinha I Gale C : Core Outcome Sets. *Arch Dis Child Educ Pract Ed.* 2018;103(3):163–166. 10.1136/archdischild-2016-312117 28667046

[10] Haywood K Whitehead L Nadkarni VM : COSCA (Core Outcome Set for Cardiac Arrest) in Adults: An Advisory Statement From the International Liaison Committee on Resuscitation. *Circulation.* 2018;137(22):e783–e801. 10.1161/CIR.0000000000000562 29700122

[11] Topjian AA Scholefield BR Pinto NP : P-COSCA (Pediatric Core Outcome Set for Cardiac Arrest) in Children: An Advisory Statement From the International Liaison Committee on Resuscitation. *Resuscitation.* 2021;162:351–364. 10.1016/j.resuscitation.2021.01.023 33515637

[12] Clarke M Williamson PR : Core outcome sets and systematic reviews. *Syst Rev.* 2016;5:11. 10.1186/s13643-016-0188-6 26792080 PMC4719739

[13] Kirkham JJ Boers M Tugwell P : Outcome measures in rheumatoid arthritis randomised trials over the last 50 years. *Trials.* 2013;14:324. 10.1186/1745-6215-14-324 24103529 PMC3852710

[14] McMillan SS King M Tully MP : How to use the nominal group and Delphi techniques. *Int J Clin Pharm.* 2016;38(3):655–62. 10.1007/s11096-016-0257-x 26846316 PMC4909789

[15] Søndergaard E Ertmann RK Reventlow S : Using a modified nominal group technique to develop general practice. *BMC Fam Pract.* 2018;19(1):117. 10.1186/s12875-018-0811-9 30021508 PMC6052560

[16] Mallett R McLean S Holden MA : Use of the nominal group technique to identify UK stakeholder views of the measures and domains used in the assessment of therapeutic exercise adherence for patients with musculoskeletal disorders. *BMJ Open.* 2020;10(2):e031591. 10.1136/bmjopen-2019-031591 32075824 PMC7044886

[17] Wallace SJ Worrall L Rose T : Which outcomes are most important to people with aphasia and their families? An international nominal group technique study framed within the ICF. *Disabil Rehabil.* 2017;39(14):1364–1379. 10.1080/09638288.2016.1194899 27345867

[18] McMillan SS Kelly F Sav A : Using the Nominal Group Technique: how to analyse across multiple groups. *Health Serv Outcomes Res Method.* 2014;14:92–108. 10.1007/s10742-014-0121-1 PMC4282516 25281284

[19] Mc Sharry J Fredrix M Hynes L : Prioritising target behaviours for research in diabetes: Using the nominal group technique to achieve consensus from key stakeholders. *Res Involv Engagem.* 2016;2:14. 10.1186/s40900-016-0028-9 29062515 PMC5611575

[20] Keegan D Heffernan E Mc Sharry J : Identifying priorities for the collection and use of data related to community first response and out-of-hospital cardiac arrest: protocol for a nominal group technique study. 2021. 10.17605/OSF.IO/VT5M7 PMC8637246 34909578

