# Supplementary material for: Identifying priorities for the collection and use of data related to community first response and out-of-hospital cardiac arrest: protocol for a nominal group technique study
Source: HRB Open Res. 2021 Nov 29;4:81. Originally published 2021 Jul 26. [Version 2] doi: 10.12688/hrbopenres.13347.2 (PMC8637246; doi:10.12688/hrbopenres.13347.2)
Supplement: Peer review history

# Peer review: Reviewer response for version 2

**DOI:** 10.21956/hrbopenres.14682.r30955

**Published:** 2021-12-01

**Authors:** Karl -Christian Thies

**Affiliations:** 1 Department of Anesthesiology, Critical Care and Emergency Medicine, Bethel Medical Centre, OWL University Hospitals, Bielefeld University, Bielefeld, Germany

Dear Authors,

 I have no further comments or questions. Good luck with the project.

 Best wishes,

 Karl

Is the study design appropriate for the research question?

Partly

Is the rationale for, and objectives of, the study clearly described?

Yes

Are sufficient details of the methods provided to allow replication by others?

Yes

Are the datasets clearly presented in a useable and accessible format?

Not applicable

Reviewer Expertise:

Cardic Arrest, First Responder Systems

I confirm that I have read this submission and believe that I have an appropriate level of expertise to confirm that it is of an acceptable scientific standard.

# Peer review: Reviewer response for version 1

**DOI:** 10.21956/hrbopenres.14535.r30771

**Published:** 2021-11-12

**Authors:** Terry P. Brown

**Affiliations:** 1 Warwick Clinical Trials Unit, University of Warwick, Coventry, UK; 2 National Institute for Health Research Applied Research Collaboration West Midlands, West Midlands, UK

This study is proposing to collect information on the work of community first responders (CFR) in Ireland using tried and tested qualitative data collection techniques.

 There is conflicting evidence about the impact CFRs can have on the likelihood of survival from out-of-hospital cardiac arrest (OHCA). Data on OHCAs is usually collected on those patients that receive treatment from EMS personnel. Little research has been published on what treatment CFRs provide and its potential impact on survival from OHCA prior to the arrival of the EMS. This is especially so for those cases that achieve a return of spontaneous circulation before EMS arrive. In addition, the role of CFRs away from the treatment of patients is not well known. The proposed study will go some way in providing essential information that could inform future policy on the roles and responsibilities of CFRs not only in Ireland but also globally.

 I found the study protocol to be clear and concise. The authors outlined their plan of study very well. I only have a couple of minor comments with regard to GDPR, and not sure whether it is HRB policy for statements to be made about where data collected is to be stored, data storage security and who will have access to this data. Also, a statement should be made that the anonymity of participants will be respected.

Is the study design appropriate for the research question?

Yes

Is the rationale for, and objectives of, the study clearly described?

Yes

Are sufficient details of the methods provided to allow replication by others?

Yes

Are the datasets clearly presented in a useable and accessible format?

Yes

Reviewer Expertise:

Out-of-Hospital Cardiac Arrest, Public Access Defibrillation, Epidemiology, Statistics

I confirm that I have read this submission and believe that I have an appropriate level of expertise to confirm that it is of an acceptable scientific standard.

# Response

**Published:** 2021-11-20

**Authors:** Dylan Keegan

**Affiliations:** National University of Ireland, Galway, Ireland

We thank the reviewer for their consideration of the protocol and valuable comments. We have addressed their concerns below and feel these changes have allowed us to improve our protocol overall. 

- **I only have a couple of minor comments with regard to GDPR, and not sure whether it is HRB policy for statements to be made about where data collected is to be stored, data storage security and who will have access to this data. Also, a statement should be made that the anonymity of participants will be respected.**

We have outlined in the 'Ethical considerations' section that this study will be conducted in accordance with NUI Galway’s University Data Protection Policy and Ireland’s General Data Protection Regulation (GDPR). However, we acknowledge that more specific information is needed regarding storage, security and access of data, as well as participant anonymity. We have added information to reflect this.

# Peer review: Reviewer response for version 1

**DOI:** 10.21956/hrbopenres.14535.r30528

**Published:** 2021-11-11

**Authors:** Karl -Christian Thies

**Affiliations:** 1 Department of Anesthesiology, Critical Care and Emergency Medicine, Bethel Medical Centre, OWL University Hospitals, Bielefeld University, Bielefeld, Germany

The authors present a study protocol, which describes a consensus process to agree on key data to collect from CFR systems and to identify barriers and facilitators to data collection. The project proposed is relevant. As the authors correctly state, data on CFR systems are scarce; recent studies on CFR systems are difficult to compare with each other because of the lack of any specific reporting standards. Therefore, it makes sense to prioritise in a first step the parameters that should be investigated.

 The suggested consensus process appears be to appropriate, provided the points below are addressed / specified:

- The size of the stakeholder groups is not clear; are there 20 per stake holder group or are there 20 individuals in total on the panel?
- If there are only 20 individuals on the panel, the patient and family perspective would be underrepresented, because the view of survivors and their families as well as the view of non-survivor families should are paramount in this process.
- How do you select the stakeholders?
- How do you ensure that long term outcome data are included into your data set?
- Do you intend to link the data to your  national cardiac arrest registry?

Is the study design appropriate for the research question?

Partly

Is the rationale for, and objectives of, the study clearly described?

Yes

Are sufficient details of the methods provided to allow replication by others?

Yes

Are the datasets clearly presented in a useable and accessible format?

Not applicable

Reviewer Expertise:

Cardic Arrest, First Responder Systems

I confirm that I have read this submission and believe that I have an appropriate level of expertise to confirm that it is of an acceptable scientific standard, however I have significant reservations, as outlined above.

# Response

**Published:** 2021-11-20

**Authors:** Dylan Keegan

**Affiliations:** National University of Ireland, Galway, Ireland

We thank the reviewer for their careful and constructive consideration of our protocol which has allowed us to make some improvements. We have addressed each of their comments below.

- **The size of the stakeholder groups is not clear; are there 20 per stake holder group or are there 20 individuals in total on the panel?**

The maximum sample size is 20 individuals in total for the panel. Previous nominal group technique (NGT) research has a range of sample sizes from 2 – 14 participants ^14,16^, with some research including larger sample sizes (24 participants) ^19^. To ensure adequate representation for each of our stakeholder groups we have opted for a larger sample size of 20 participants. The Participants and recruitment section has been revised to more clearly state this.

- **If there are only 20 individuals on the panel, the patient and family perspective would be underrepresented, because the view of survivors and their families as well as the view of non-survivor families should are paramount in this process.**

We agree that ensuring the patient and family perspective is appropriately represented is essential and have taken this into consideration in planning the consensus process. We have updated the Participants and recruitment section to reflect this.

- **How do you select the stakeholders?**

A purposeful sampling strategy will be used to select representatives from various stakeholder groups. Peer consultation will be employed by the research team and the Scientific Advisory Group associated with this study to identify suitable participants from each stakeholder group who have relevant experience and expertise. We have now included this point in the Participants and recruitment section.

- **How do you ensure that long term outcome data are included into your data set?**

We have now addressed this point in the Design section and the Discussion section of the protocol. When asking participants for suggestions in the online survey and consensus process, we will not specify a short or long term timeframe for the outcome data, as such there will be an open timeframe. This is because the nominal group technique requires that data generation and prioritization is participant-driven, rather than researcher-driven, such that it is the stakeholders who create and determine the priorities for data collection and use. We recognize that long term outcome data are important but that there is a risk that they may not be identified as a priority by the stakeholders. However, the purposeful sampling strategy of this study means that a range of participants with diverse expertise will be recruited, including researchers, clinicians, and patients/families. It is anticipated that the diversity of the sample will help to ensure that all key perspectives are represented and that a wide array of priorities are generated, including priorities related to both short and long term outcome data.

- **Do you intend to link the data to your national cardiac arrest registry?**

Yes, the data collected will be included in the National Out-of-hospital cardiac arrest register (OHCAR) and in the OHCAR annual report. This has now been outlined in the Plans for dissemination section and in the Discussion section.
